# Cognitive Video Surveillance Management in Hierarchical Edge Computing System with Long Short-Term Memory Model

**DOI:** 10.3390/s23052869

**Published:** 2023-03-06

**Authors:** Dilshod Bazarov Ravshan Ugli, Jingyeom Kim, Alaelddin F. Y. Mohammed, Joohyung Lee

**Affiliations:** 1 Department of Computing, Gachon University, Seongnam-si 13120, Republic of Korea; 2 Advanced Research Team, NHN Cloud Corp., Seongnam-si 13487, Republic of Korea

**Keywords:** LSTM, cognitivevideo surveillance management, hierarchical edge computing, deep learning, object detection and tracking

## Abstract

Nowadays, deep learning (DL)-based video surveillance services are widely used in smart cities because of their ability to accurately identify and track objects, such as vehicles and pedestrians, in real time. This allows a more efficient traffic management and improved public safety. However, DL-based video surveillance services that require object movement and motion tracking (e.g., for detecting abnormal object behaviors) can consume a substantial amount of computing and memory capacity, such as (i) GPU computing resources for model inference and (ii) GPU memory resources for model loading. This paper presents a novel cognitive video surveillance management with long short-term memory (LSTM) model, denoted as the CogVSM framework. We consider DL-based video surveillance services in a hierarchical edge computing system. The proposed CogVSM forecasts object appearance patterns and smooths out the forecast results needed for an adaptive model release. Here, we aim to reduce standby GPU memory by model release while avoiding unnecessary model reloads for a sudden object appearance. CogVSM hinges on an LSTM-based deep learning architecture explicitly designed for future object appearance pattern prediction by training previous time-series patterns to achieve these objectives. By referring to the result of the LSTM-based prediction, the proposed framework controls the threshold time value in a dynamic manner by using an exponential weighted moving average (EWMA) technique. Comparative evaluations on both simulated and real-world measurement data on the commercial edge devices prove that the LSTM-based model in the CogVSM can achieve a high predictive accuracy, i.e., a root-mean-square error metric of 0.795. In addition, the suggested framework utilizes up to 32.1% less GPU memory than the baseline and 8.9% less than previous work.

## 1. Introduction

The number of Internet of things (IoT) devices that have been widely used for video surveillance services (e.g., IoT cameras) is expected to nearly treble, from 9.7 billion IoT devices in 2020 to more than 29 billion IoT devices in 2030 [1]. Deep learning (DL)-based video surveillance services have been widely spread throughout industries in recent years due to the democratization of artificial intelligence (AI), as exemplified in object detection, crime prevention, protection of a process, autonomous driving, protecting neighborhoods, patient monitoring, and video surveillance systems [2]. Moreover, in DL-based video surveillance, due to the limited computing capability of IoT devices, it requires the support of a nearby edge computing server that can automatically identify objects and support the observation of the object’s movement with a contextual purpose (e.g., abnormal behavior detection in public or private places, patient monitoring in healthcare service). Nevertheless, such DL-based video surveillance, including object recognition and motion tracking tasks, needs a large quantity of GPU processing power and GPU memory, which increases operating costs at the edge computing server as the number of IoT cameras handled increases.

This paper proposes a novel cognitive video surveillance management with a long short-term memory (LSTM) model [3], denoted as the CogVSM framework. We consider the DL-based video surveillance services in hierarchical edge computing systems, which require object movement and motion tracking (for detecting abnormal object behaviors, for instance), consuming a sizable quantity of computing and memory resources, such as (i) GPU computing resources for the model inference, and (ii) GPU memory resources for loading the model. The suggested CogVSM includes forecasts of object appearance patterns and updating the threshold time value via smoothing out the LSTM-based model forecasts by utilizing the EWMA technique that is needed for adaptive model release, specifically to lessen standby GPU memory due to the rarely occurring abnormal object behaviors for delivering DL-based video surveillance services in an efficient manner. Here, we want to reduce the amount of standby GPU memory while preventing needless model reloads for unexpected object appearance. Thus, such GPU memory reduction can enable the deployment of deep learning (DL)-based video surveillance over edge computing servers in a cost-effective manner by reducing the hardware requirements and associated costs and bringing the benefits of advanced machine learning algorithms to a wider range of use cases and applications.

The CogVSM framework archives the object appearance pattern prediction by training earlier time-series patterns. The proposed CogVSM framework dynamically regulates the threshold time value using EWMA by referencing the outcome of LSTM-based prediction, which causes the model to release if the object is recognized within the time limit. The following is a summary of the specific contributions of this study:

- We design a novel cognitive video surveillance management system with an LSTM model for a cost-effective video surveillance system.
- We consider hierarchical edge computing systems by determining whether objects are detected or not in the video frames using the YOLO algorithm for the first-level edge.
- Our suggested framework dynamically controls the threshold time of object occurrence for the second-level edge by utilizing the detection info from the first-level edge to reduce such a standby GPU memory by a model release and to prevent latency during needless model reloading for an unexpected object appearance.
- The proposed CogVSM uses an LSTM model for predicting future object appearance patterns and controlling the threshold module hierarchically.
- The LSTM model makes predictions based on historical object occurrence patterns. Considering the outcomes of the LSTM prediction, the controlling threshold module utilizes an exponential weighted moving average (EWMA) technique for smoothing the LSTM prediction result that triggers the model release.
- If there is no appearance of an object during that threshold time, the AI model is released, and methods for reducing the GPU memory are given based on the LSTM prediction model and regulating threshold module.

The rest of this paper is structured as follows. In Section 2, related works to our work are provided. The background of LSTM model is explained in Section 3. The suggested framework is outlined in Section 4. Section 5 discusses the framework’s implementation. The evaluation results are shown in Section 6. Finally, Section 7 brings the paper to a conclusion.

## 2. Related Work

Recently, several approaches were proposed for enabling DL-based video surveillance assisted by an edge computing server cost-effectively. For instance, refs.  [4,5,6,7,8] made significant achievements in energy efficiency through edge computing and optimizing mechanisms, resulting in a reduced network bandwidth and response time in IoT-based smart video surveillance systems for effective object detection and abnormal behavior analysis. Moreover, ref. [9] demonstrated an effective edge-computing-based architecture for an unmanned aerial vehicle (UAV) environment to minimize delay and network traffic consumption by identifying objects’ anomalous occurrences. The suggested study tried to filter video frames of interest at the edge device by transmitting only the video frames that should be analyzed for inference to the cloud server. Authors in [10] suggested a method to detect objects and track the object of interest accurately compared to traditional methods while addressing the GPU processing power reduction and motion tracking accuracy. However, those studies did not consider the hierarchy of edge computing systems and the practice used for video surveillance services.

More recently, ref. [11] proposed a platform for monitoring road construction safety using UAV by detecting and tracking constructors. Ref. [12] introduced a stable and effective object tracking system called video analytics edge computing (VAEC) by adopting a tracking-by-detection (TBD) method, which provided a real-time increased context awareness for human detection in video surveillance. Ref. [13] suggested a queue control-based object tracking technique that managed the maximal queue size dynamically to fulfill the target latency of the real-time intelligent crossing detection system. Simultaneously, the video frame was sent in the suggested queue to request for detecting and tracking objects. Several efforts in [14,15,16,17] on anomalous activity monitoring were based mostly on low-level features. However, all works above considered a policy of always loading a heavy DL model that tracked the movement of an object in the DL-based video surveillance system, which results in unnecessary GPU memory consumption when there is no object appearance. Interestingly, ref. [18] revealed that if there was no appearance of an object during a specified threshold time, the heavy DL model could be released to save unnecessary GPU memory consumption. Even though [18] achieved a performance superior to other approaches in terms of GPU memory consumption reduction, an unnecessary delay when reloading the model might happen depending on the corresponding hyperparameter of the threshold time. For example, if the threshold time value was too large (e.g., 30 s), then the release frequency of the DL model decreased and consumed more GPU memory, whereas if the threshold time value was too small (e.g., 10 s), a DL model release and reload switching frequently occurred, causing reloading delays.

## 3. LSTM Background

**Figure 1:**
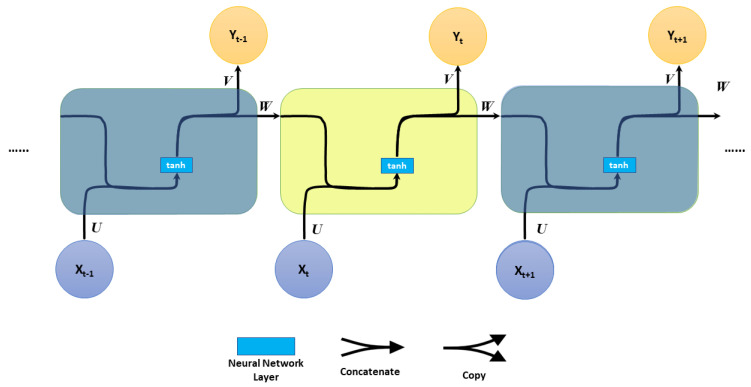
RNN architecture.

RNNs are artificial neural networks in which the connections between processing units form a directional circle [19], as shown in Figure 1.

The fundamental formulas that regulate the calculation in an RNN are:

(1) $$\begin{array}{cc} h_{t} & =f(Ux_{t}+Wh_{t-1})\\ y_{t} & =softmax(Vh_{t}) \end{array}$$

where *f* is an activation function (i.e., *tanh* function), $X_{t}$ and $Y_{t}$ are the input and the output vectors at time step *t*, and $h\in R^{N}$ is the hidden-layer state with *N* hidden units at time step *t*. *U*, *W*, and *V* are the weight parameters.

RNNs can process sequential data [20] (e.g., speech recognition, handwritten books, machine translation, and NLP). However, RNNs have several drawbacks that can make them challenging to use in practice. First, to forecast the present result, a reference to some particular information saved long ago is very important. However, RNNs are unable to store information for a more extended time, also known as the “long-term dependencies” problem. Second, RNNs also lack a forget gate. A forget gate is a mechanism that allows the network to discard information from previous inputs selectively. This can be useful for preventing the network from becoming overwhelmed with irrelevant information. Other issues with RNNs are that they can also suffer from problems with exploding or vanishing gradients. Specifically, the gradients are utilized to adjust the neural network weights during the training process. If the gradients are too large, the network may overshoot its target and fail to converge. On the other hand, if the gradients are too small, the network may not learn at all.

LSTM [3] is a kind of recurrent neural network (RNN) designed to address the aforementioned drawbacks of traditional RNNs. Specifically, LSTM can effectively deal with long-term dependencies, the lack of a forget gate, and problems with exploding or vanishing gradients. There is no requirement to retain a finite number of states from the beginning with LSTM, as is necessary for the hidden Markov model (HMM) [21]. The complication of adjusting each weight is decreased to O(1) with LSTM, which is comparable to backpropagation through time (BPTT) [22].

**Figure 2:**
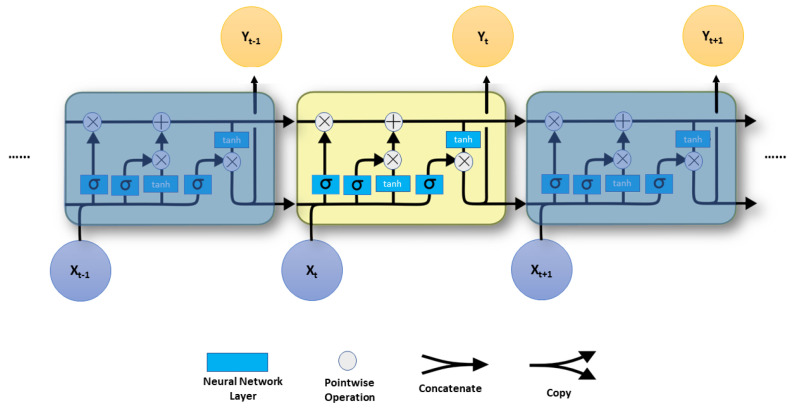
Long short-term memory architecture.

The LSTM unit was used in this paper as shown in Figure 2.

Instead of just one, four neural network layers connect in a special manner in LSTM. All of the preceding elements are the same size as the hidden vector. In other words, LSTM contains an input gate, forget gate, output gate, and a memory cell in addition to a hidden layer. Every line shown in Figure 2 transmits a whole vector from one node’s output to another node’s input. The secret to LSTM is the horizontal line that goes through the top of the figure and indicates the cell state. In some aspects, the cell state is similar to a conveyor belt. Information goes immediately down the entire chain with only a few modest linear interactions. Information can easily continue to flow unaltered along it. The blue boxes represent learned neural network layers, and the ice blue circles represent pointwise operations such as vector addition and multiplication. Merging lines are concatenations, but forking lines are their content that has been copied and is being transmitted to other destinations.

We also provide the equations of LSTM for a single memory unit only using the following equations:

(2) $$\begin{array}{cc} f_{t} & =\sigma (W_{f}\cdot \left[h_{t-1},x_{t}\right]+b_{f}),\\ i_{t} & =\sigma (W_{i}\cdot \left[h_{t-1},x_{t}\right]+b_{i}),\\ {\hat{c}}_{t} & =f\sigma (W_{c}\cdot \left[h_{t-1},x_{t}\right]+b_{c}),\\ c_{t} & =c_{t-1}\cdot f_{t}+i_{t}\cdot {\hat{c}}_{t},\\ o_{t} & =\sigma (W_{o}\cdot \left[h_{t-1},x_{t}\right]+b_{o}),\\ h_{t} & =o_{t}\cdot tanh\left(c_{t}\right), \end{array}$$

where $f_{t}$ presents the forget gate, σ(x) is the activation function (i.e., logistic sigmoid function), and $tanh(c_{t})$ is also the activation function (i.e., hyperbolic tangent function), $i_{t}$ is the input gate, $c_{t}$ is the input vector for cell states, $c_{{\hat{c}}_{t}}$ is the input temporary vector for cell states, and $o_{t}$ is the output gate. $b_{i}$, $b_{f}$, $b_{o}$, and $b_{c}$ are the bias terms.

## 4. Cognitive Video Surveillance Management (CogVSM) Framework

**Figure 3:**
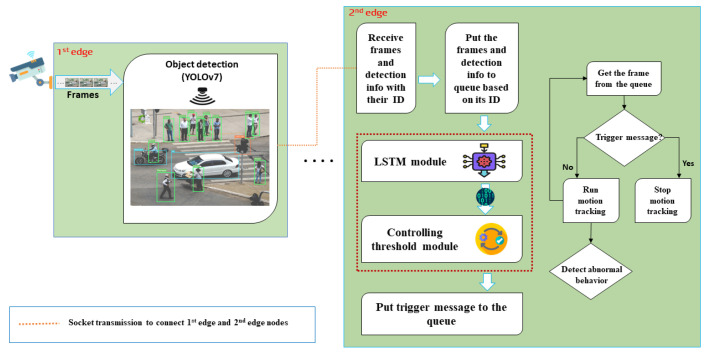
Overall architecture of the proposed framework.

In this section, we first present the suggested CogVSM framework, depicted in Figure 3 as the entire approach. Our main contribution in CogVSM is divided into two parts that are highlighted with a red dashed rectangle at the 2nd edge node.

- **LSTM**: The long short-term memory (LSTM) module predicts future object occurrence using earlier time-series patterns. Then, the LSTM module transmits the predicted values to the controlling threshold module.
- **Controlling threshold module**: The controlling threshold module receives object occurrence prediction outcomes. Then, the exponential weighted moving average (EWMA) is calculated to update the threshold time value continuously.

As shown in Figure 3, the CogVSM framework is separated into two edge nodes: a 1st edge node (including the task of object detection) and a 2nd edge node (including the tasks of predicting future object occurrence (i.e., LSTM module), the controlling threshold module, and motion tracking module). We assume the 1st and 2nd edge nodes are connected to each other. Notably, the processes start taking input video frames from the attached IP camera in the 1st edge node, and then the following four tasks are performed:

1. Task (1): Running the object detection module. When the input video frames arrive, the YOLO object detection algorithm [23], task (1), is activated. As soon as objects are detected, the 1st edge node transmits the detection info (i.e., detected number of people and time) and image frames to the 2nd edge for further process.
2. Task (2): For object movement and motion tracking, the frames containing the object detection results (such as human bounding boxes) and detection info are delivered to the 2nd edge node. When the video frames and detection info with their ID are received at the 2nd edge node from the 1st edge node, the frames and detection info are placed in a process queue (based on their ID). Then, the detection info is transmitted to the LSTM module to predict future object occurrence. Task (2) predicts how many objects will occur in the future and transmits the prediction values to the controlling threshold module. After that, the controlling threshold module receives the predicted data.
3. Task (3): Controlling threshold module. In this module, an exponential weighted moving average (EWMA) is calculated based on whole sequences of numbers to simultaneously update the threshold time value. Finally, using a threshold, the controlling threshold module decides whether to send stop instruction or video frames to the motion tracking module, which is the next task.
4. Task (4): Motion tracking module. If the control module decides to release a DL-based motion tracking model, a trigger signal transmits the signal to the motion tracking model with a halt instruction. Otherwise, video frames are delivered to the motion tracking model with the queue. Task (4) executes object movement and motion tracking whenever the video frame arrives through the control module.

Here, we introduce Algorithm 1, which represents how the motion tracking module is released based on queue and threshold time value. In this case, the control module decides either to release the DL-based motion tracking module or not by comparing the empty queue value and threshold time value.
**Algorithm 1** CogVSM.**Input:** 
Threshold for motion tracking $\theta_{m}$, window size $W_{n}$, empty queue $t_{empty}$1:**while ***True ***do**2:    Put received frames and detected number of objects into the queue3:    Calculate EWMA value based on prediction results of LSTM module4:    Update $\theta_{m}$ based upon the EWMA value5:    **if** $t_{empty}$ ≥ $\theta_{m}$ **then**6:        Deliver a trigger signal to the motion tracking module along with a halt instruction.7:    **else**8:        Deliver the obtained frames to the motion tracking procedure through queues.9:    **end if**10:**end while**

### 4.1. Long Short-Term Memory (LSTM) Module

The main objective of this module (by only adopting the LSTM model) is to predict the future object occurrence by utilizing the detected number of objects from the 1st edge node, then transmit forecast values to the controlling threshold module, which coincides with the task that determines the prediction of whether the object will occur or not. This can help us to forecast future object occurrence in advance, so that we can control the threshold time value for an adaptive model release and prevent the model reloading latency because of sudden object occurrence. As a result, we can achieve a sufficient GPU memory usage reduction by predicting future object occurrence via our LSTM model. Notably, our LSTM-based model is trained with the historical detected numbers of object and gives results in non-negative numbers as usual.

### 4.2. Controlling Threshold Module

The controlling threshold module consists of mainly two parts: (A) the first part starts by calculating the EWMA value as shown in (Equation (3)) and (B) the second part updates the threshold time value by comparing EWMA values. The EWMA is a weighted moving average (WMA) technique that gives more weight or importance to recent data. EWMA is better at recognizing chart changes and trends, which was first introduced by [24]. Similar to the simple moving average (SMA), the EWMA technique is used to observe data trends over time and watch several EWMAs simultaneously, which is easy to do with moving average ribbons. However, compared to the simple moving average (SMA) technique, the EWMA technique gives a greater weight to recent (more relevant) data [24].

The EWMA technique produces weighted average data, where we have greater control over the weighted moving average than the SMA and EMA techniques. Typically, the more recent data points are given a greater weight.

Below, we provide the recursive formula for the EMA:

(3) $$\begin{array}{c} V_{t}=\left(1-\beta \right)*V_{t-1}+\beta *W_{t} \end{array}$$

where $V_{t}$ represents the current EWMA value, $V_{t-1}$ represents the previous EWMA value, $W_{t}$ represents the current data point, and β represents a constant (or hyperparameter) value between 0 and 1. When we calculate the EWMA, we use the EWMA value in Algorithm 2.
**Algorithm 2** Exponential Weighted Moving Average (EWMA).**Input:** 
Motion tracking threshold $\theta_{m}$, EWMA ($V_{n}$)1:**while ***True ***do**2:    Calculated EWMA ($V_{n}$)3:    **if** $V_{n}$ > 0.5 **then**4:        Motion tracking threshold is increased by 1 sec ($\theta_{m}$←$\theta_{m}$ + Δ, $\theta_{max}$)5:    **else**6:        Motion tracking threshold is decreased by 1 sec ($\theta_{m}$ ← $\theta_{m}$ - Δ, $\theta_{min}$)7:    **end if**8:**end while**

## 5. Implementation

We considered two edge nodes in our test scenario: first edge node and second edge node. These edge nodes were connected to each other. The Jetson Nano [25] (integrated with an ARM A56 CPU and NVIDIA Maxwell GPU) was used to implement the first edge node. (We found that the motion tracking outcomes of the Jetson Nano ranged between 1.2 to 3 frames per second based on standard measure testing, which was too slow for real-time security monitoring. Therefore, we used a hierarchical edge computing system in which motion tracking was performed on the 2nd edge node after an object detection on the first edge node.) One IP camera connected via USB connection was installed on the first edge. For example, the GeForce RTX 2080 SUPER GPU was more potent than the GPU on the second edge node.

**First edge node:** The first edge node communicated with the second edge node and contained the object detection. In order to detect objects, we employed the YOLO model. In particular, the first edge node used YOLOv7-tiny [26], a relatively tiny model for limited mobile and edge devices. Using Python sockets, the first edge node also sent video frames to the second edge node.

**Second edge node:** The second edge node featured the prediction of future object occurrence (i.e., LSTM [19]), the controlling threshold module, motion tracking module, and a connectivity with the first edge node. We trained the LSTM model to predict future object occurrence from historical object pattern data. The controlling threshold module updated the threshold value, which halted the process to free up GPU memory capabilities. The Python Process class and Python Thread class were utilized to implement the motion tracking module and handle the data through both edge nodes. As previously stated, the motion tracking and communication processes were implemented in the control thread. The second edge node first accepted frames with messages via a Python socket from the first edge node. Then, the frames were put into queues to enable communication with the object prediction and motion-tracking algorithms. A threshold value was set for a certain amount of time (i.e., seconds) when there were no frames in the queue. After that, the controlling threshold module updated the threshold value based on the LSTM prediction. If the empty queue value exceeded the updated threshold time value, a trigger signal with stop instruction was sent to the queue, and the motion-tracking process was terminated. We utilized the TF-pose-estimation [27] (a TensorFlow-based human pose estimation system) for motion tracking.

**Figure 4:**
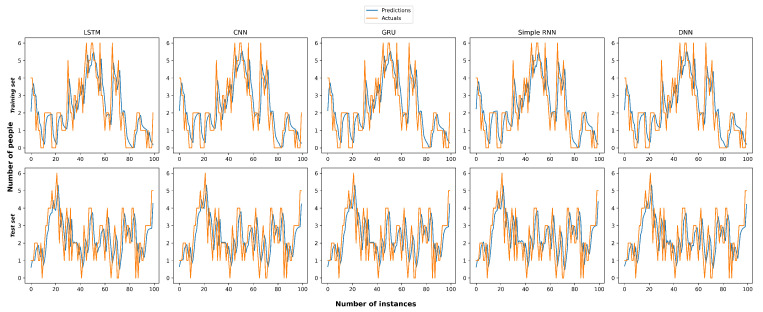
The prediction error measurement of mainstream deep learning models on training and test sets.

Since our goal was to predict object occurrence in a video surveillance service as a time series prediction problem, we evaluated commonly used deep learning models for time series prediction [28,29]. We decided to choose the RMSE metric [30] to measure the error of the deep learning model predictions as we could penalize larger errors [31], because we sometimes had larger prediction errors because of sudden object occurrence in the video surveillance services. We experimented with long short-term memory, convolutional neural networks [32,33], gated recurrent units [34], simple recurrent neural networks [19,35] and deep neural networks on the same dataset in Table 3, and the results in Figure 4 and the accuracy loss in terms of root-mean-square error [30] are given in Table 1.

Our experiments reveal that the LSTM model surpassed other common deep learning models with an RMSE loss accuracy of 0.795.

In our simulation, we ran the proposed LSTM model on TensorFlow 2.9.0 [36] API under Python 3.9 [37]. With Keras library [38], we evaluated our proposed LSTM performance using the root-mean-square error (RMSE). Moreover, we initialized some hyperparameters (e.g., optimizer, learning rate, loss). These hyperparameters controlled the characteristics of the LSTM network to provide the best prediction. Table 2 describes the training hyperparameters of the proposed LSTM network in detail.

We collected a dataset using the Shinjuku Kabukicho live cam [39] in Tokyo, Japan, via Yolo v7-tiny [26] to train our LSTM model. This dataset describes the number of people who appeared on a closed-circuit television (CCTV) video every second for two days. Table 3 shows the dataset description.

**Figure 5:**
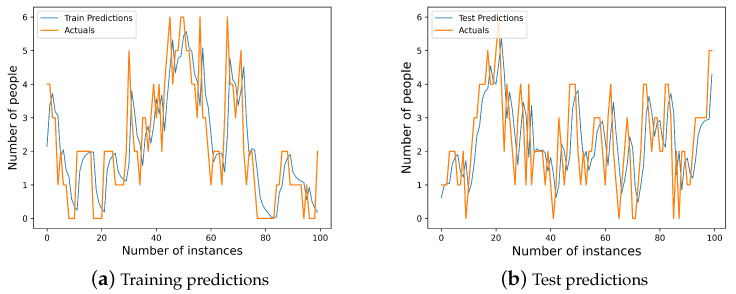
The implementation results on 1st edge node and 2nd edge node.

Figure 5 presents the implementation results of the LSTM model. Specifically, Figure 5a,b show the average of every second forecast obtained by the LSTM in terms of predicting results in the training and test sets, respectively. It can be seen that the model fit extremely well on the whole interval of 100 s in this case.

## 6. Performance Evaluation

This section includes measurement-based performance evaluations to illustrate the viability of the suggested CogVSM system. Here is a quick summary of the benchmark, the previous work, and the suggested structure for the evaluation part:

- **Benchmark**: The method of continuous motion tracking without the suggested CogVSM was used for the evaluation. This standard was referred to as baseline. We also evaluated our work with previous work, namely, AdaMM. [18].
  - **Baseline**: For a precise evaluation, the baseline included hierarchical object recognition and motion tracking, according to the same structural design as the suggested CogVSM framework. In that case, when the motion tracking model was loaded for observing motion, the baseline kept holding GPU memory for tracking motion despite the absence of an object in the video frame.
  - **AdaMM**: All suggested modules (i.e., frame differences and management of adaptive processes) were included in AdaMM [18].

- **Proposed CogVSM**: This covered all suggested modules, such as the LSTM prediction and controlling threshold module.

**Figure 6:**
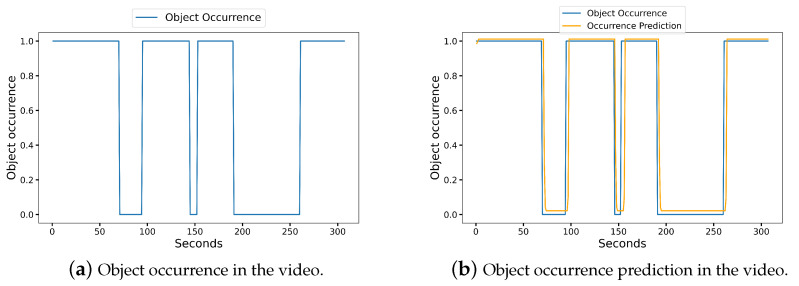
Object occurrence in the sample video and its pretrained LSTM-based prediction.

We used the commercial edge device Jeston Nano at the first edge node for thoroughly measuring our system. It had a Maxwell GPU with 128 cores. We utilized a desktop with a GeForce RTX 2070 SUPER for the second edge node. Additionally, to imitate the case of periodic object appearance in surveillance videos, we generated one example video. The video duration, frame rate, and resolution values were just about 300 s, 30 fps, and 1280 × 720, respectively. Figure 6a shows the range of object presence in the video and Figure 6b shows the forecast of object occurrence. Therefore, the values of one and zero revealed the object was either detected or undetected utilizing our YOLO algorithm at the first edge node. In the video, the object was only detected during the intervals [0 s, 70 s], [94 s, 145 s], [152 s, 190 s], and [261 s, 307 s]. However, there was no object in other intervals because the object was not detected.

We evaluated only one crucial performance indicator obtained from the second edge node, that is, the GPU memory utilization, denoted as $GPU_{m}$. The range of $GPU_{m}$ was [0%, 100%]. Table 4 provides a summary of the parameters and settings of the proposed framework.

About 3600 megabytes (MiB) (total memory 7979 Mib) were needed to load the model for motion tracking; hence, $GPU_{m}$ was roughly 46%. Moreover, $\theta_{m}$ was the threshold time value (e.g., $\theta_{m}$ = 10 s and 30 s).

### 6.1. Evaluation of the Suggested Work

**Figure 7:**
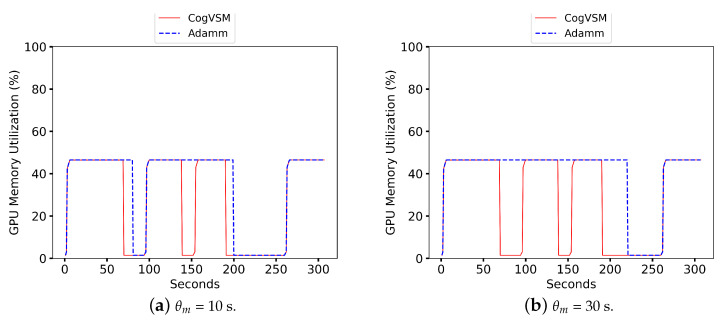
Comparison of Proposed CogVSM framework and AdaMM framework in terms of GPU memory utilization on 2nd edge node.

In this subsection, we compare our suggested work with previous work. Figure 7 shows the performance comparison of the suggested CogVSM framework to AdaMM [18]. Here, the detection process was performed on the sample video at the first edge, and the detected videos were sent through Python sockets to the second edge node. Specifically, at 0 s, both frameworks loaded models for the video request. However, the proposed framework utilized less GPU memory than the AdaMM framework for all $\theta_{m}$, i.e., the time value for the terminating process. This was due to the suggested work of releasing the GPU memory for motion tracking based on the LSTM prediction and controlling the threshold very sensitively. Still, the AdaMM framework [18] released it based on $\theta_{m}$ s values. For a precise evaluation, we tested the AdaMM framework with constant $\theta_{m}$ values (i.e., $\theta_{m}=10$ s and $\theta_{m}=30$ s) as in Figure 7.

The GPU memory at the second edge node was utilized efficiently by predicting object occurrence and controlling forecast outcomes; meanwhile, AdaMM used more GPU memory by waiting for frames till $\theta_{m}$ s and the amount of memory varied according to the $\theta_{m}$ values as in Figure 7. When $\theta_{m}=10$ s (Figure 7a), the suggested framework terminated the motion tracking process three times by releasing the GPU memory; however, AdaMM released the GPU memory twice, which meant that the proposed framework was working effectively with the prediction and controlling module. The second edge node did not receive any frame from the first edge node ($\theta_{m}$ value) at approximately 70 s and 94 s. As soon as the second edge received frames from the first edge node, at roughly 95 s, the suggested framework loaded up the model for observing motion. When $\theta_{m}=30$ s (Figure 7b), AdaMM released the GPU memory just once, while the proposed CogVSM stopped the procedure three times.

### 6.2. Performance of the Proposed CogVSM Model in Urban and Rural Area

To validate the performance of our proposed CogVSM in urban area and rural areas, we compared the suggested framework with the AdaMM framework. We tested both frameworks using two different sample videos from the Shinjuku Kabukicho live cam [39] in Tokyo, Japan, for the urban area and the Koh Samui live cam [40] in Chaweng, Thailand, for the rural area. In the sample video from [39], objects occur approximately during the intervals [0 s, 35 s], [43 s, 106 s], [132 s, 139 s], [142 s, 206 s], [245 s, 305 s] and in the sample video from [40], objects occur in the intervals [0 s, 51 s], [127 s, 169 s], [225 s, 234 s], respectively.

**Figure 8:**
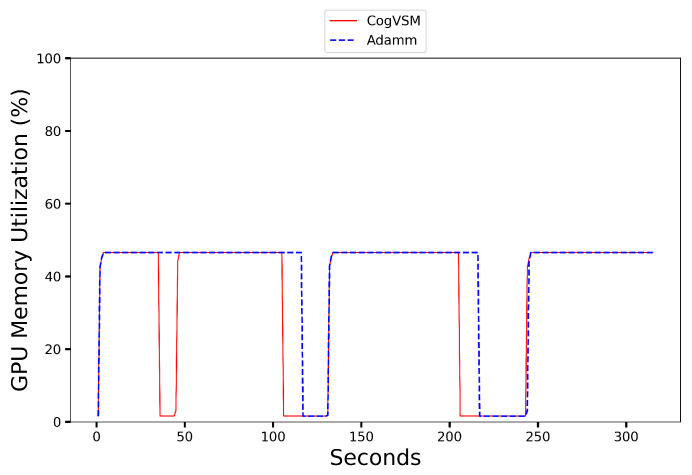
GPU memory utilization on an urban area video of the proposed CogVSM framework compared to the AdaMM framework ($\theta_{m}=10$ s for AdaMM).

**Figure 9:**
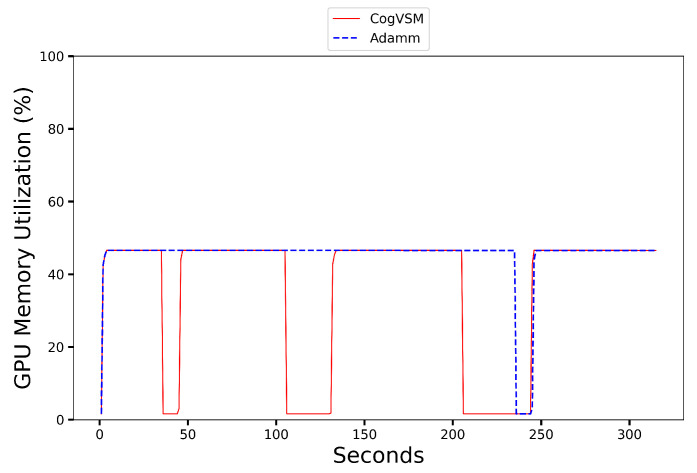
GPU memory utilization on an urban area video of the proposed CogVSM framework compared to the AdaMM framework ($\theta_{m}=30$ s for AdaMM).

**Figure 10:**
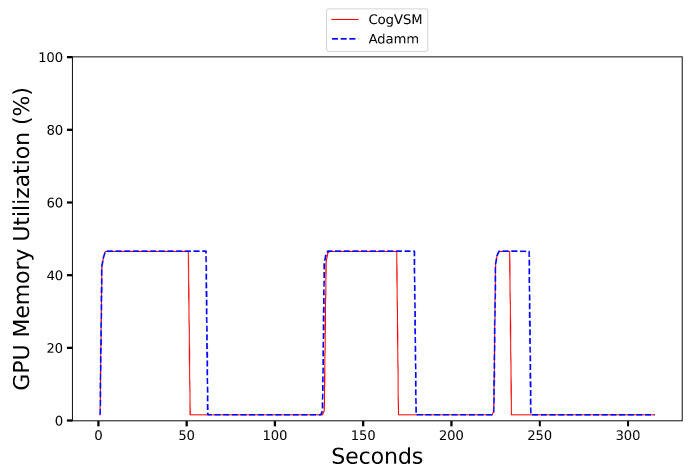
GPU memory utilization on a rural area video of the proposed CogVSM framework compared to the AdaMM framework ($\theta_{m}=30$ s for AdaMM).

**Figure 11:**
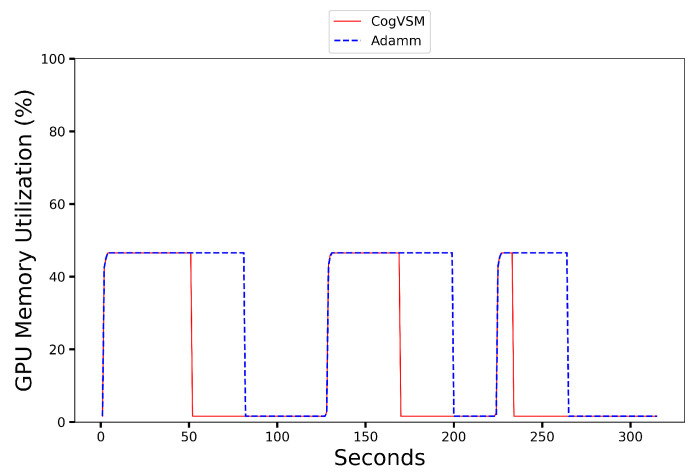
GPU memory utilization on a rural area video of the proposed CogVSM framework compared to the AdaMM framework ($\theta_{m}=30$ s for AdaMM).

Figure 8, Figure 9, Figure 10 and Figure 11 present the results where despite the object occurrence in urban areas being relatively higher than in rural areas, it was nearly impossible to release the DL-based model for motion tracking; however, our proposed framework adaptively released the DL model by smoothing out short-term fluctuations and highlighting longer-term trends of LSTM’s future object occurrence forecasts. In Figure 8, even our LSTM model predicted that there was no object during [139s, 142s] in the sample video for the urban case; CogVSM did not release the DL-based model and avoided the reloading model delay as the EWMA controlled the transient object occurrence. However, when we set $\theta_{m}=30$ s for AdaMM, AdaMM released the DL-based motion tracking model just once in Figure 9 because of the longer threshold time value imposed, and it consumed more GPU memory.

Moreover from Figure 10 and Figure 11, we witnessed that CogVSM framework consumed less GPU memory than the AdaMM framework even in the rural area case. However, the GPU memory utilization of AdaMM increased as we increased the $\theta_{m}$ value.

Additionally, the proposed CogVSM adaptively managed such $\theta_{m}$ value to achieve the aforementioned performance while preventing model reloading delay. However, AdaMM was unstable and varied with respect to the setting of $\theta_{m}$. The constant $\theta_{m}$ value resulted in unnecessary transition and delay when there was a sudden object for a short-term duration (e.g., [1 s, 3 s]) in a video and held more GPU memory. The proposed CogVSM minimized such unnecessary transitions and delays. Furthermore, properly training the LSTM model in the case of rural or urban areas gave a relatively better performance which depended on the application that went beyond the purpose of our research.

### 6.3. The Impact of Exponential Weighted Moving Average (EWMA) on Model Reloading Latency

To represent the impact of the EWMA technique on the model reloading latency, we tested our suggested framework on a sample video, and the results are shown in Table 5. The actual values illustrate the detected number of people at the first edge node. Based on actual values, the LSTM-based model predicted the number of people, and the EWMA value was calculated to update the $\theta_{m}$ value at the second edge node. Based on our experimental results, we decided to choose the value for the threshold time value as $\theta_{m}\in$ [0 s, 2 s] Here, it is obvious that the suggested CogVSM framework using the EWMA technique avoided the reloading model latency (i.e., about 3 s) because of the sudden object absence for a short time. Notably, the EWMA technique allowed the CogVSM framework to control LSTM-model-based forecasts and adjust the $\theta_{m}$ value dynamically as the EWMA smoothed out object occurrence prediction.

Moreover, frequently reloading the model produced an overhead which could cause additional latency. However, the suggested approach made a trade-off between latency and GPU memory savings by preventing model reloading, even if there was a sudden object occurrence in a video.

## 7. Conclusions

In this paper, we developed the CogVSM framework, which adaptively conducted object detection and motion tracking in a hierarchical edge computing system to minimize GPU memory consumption. These GPU memory reductions are extremely valuable for allowing additional AI services on edge computing servers with finite GPU memory access. Moreover, by reducing the memory requirements of deep learning algorithms, we can use them on smaller and more affordable edge computing servers, which can bring the benefits of advanced video surveillance to a wider range of applications. We evaluated the performance of our framework on commercially available edge devices (i.e., Jetson Nano). Based on precise measurement performance evaluation, the suggested CogVSM consumed up to 32.1% less GPU memory than the existing baseline solution and 8.9% less than AdaMM. Furthermore, we tested the performance of our framework for the urban and rural cases. In both cases, our work surpassed and saved more GPU memory than AdaMM. Interestingly, both proposed CogVSM and AdaMM frameworks obtained the best increase in performance when object occurrence was rarely detected. Importantly, our suggested framework achieved better performance and saved GPU memory compared to AdaMM.

However, like any DL-based framework, CogVSM has limitations. Specifically, the framework’s ability to predict object occurrence may be limited in certain scenarios, such as cases with highly variable or unpredictable object movement patterns, as we only tested in urban and rural cases. As such, further research and development may be needed to improve the framework’s performance and enhance its predictive capabilities with valuable, diverse, and representative data in these challenging scenarios. Additionally, ongoing improvements in hardware technology and software algorithms may provide new opportunities to optimize the CogVSM framework and enhance its capabilities. Ultimately, ongoing research and development in this area will be critical to unlocking the full potential of edge computing for video surveillance and other applications and to enable more efficient and effective use of limited hardware resources in the edge computing ecosystem. In our future work, we will expand CogVSM using a federated learning framework for efficient training through distributed edge servers. Furthermore, by applying an experience-driven algorithm (e.g., reinforcement learning), the threshold value ($\theta_{m}$) can be adjusted more intelligently.

## Figures and Tables

**Table 1:** The prediction error measurement of the mainstream deep learning models in terms of root-mean-square error metric.

Models	RMSE Value
Long short-term memory	0.7950
Convolutional neural networks	0.7997
Gated recurrent units	0.7980
Simple recurrent neural networks	0.8002
Deep neural networks	0.7982

**Table 2:** Training hyperparameters.

Hyperparameter	Value
Optimizer	Adam
Loss	MeanSquaredError
Data preprocessing (normalization)	MinMaxScaler
Learning rate, (α)	0.001
Epochs	200
RootMeanSquareError (RMSE)	0.7950
Batch size	64

**Table 3:** Dataset description.

Parameters	Value
Dataset characteristics	Multivariate,Time−Series
Number of attributes	2,(time,number *of people)*
Attribute characteristics	Categorical,Integer
Missing values?	No
Number of instances	172,000

**Table 4:** Configuration and hyperparameter of the 2nd edge node.

Parameter	value
GPU memory usage, $GPU_{m}$ (%)	[0%, 100%]
Threshold for stopping the process, $\theta_{m}$ (s)	10 s, 30 s

**Table 5:** The result of CogVSM on a simple video.

Actual	Prediction	EWMA	$\theta_{m}$
2	1.256708	0	2
1	0.608845	1.131037	2
0	0.371257	0.547960	1
0	0.888697	0.447235	2
1	2.173102	0.854624	2
3	2.649452	2.000515	2
3	2.199124	2.469969	2
2	0.922963	2.179263	2
0	1.797102	1.077663	2
2	2.518532	1.835318	2
3	1.574680	2.374445	2
1	0.681114	1.600744	2
0	0.408341	0.850447	2
0	0.268634	0.527581	1
0	0.803959	0.326815	2
1	1.559714	0.776321	2
2	3.114499	1.436424	2
4	2.349476	2.880681	2
2	1.590056	2.258170	2
1	0.721493	1.719118	2
0	0.427102	0.875161	2
0	0.256033	0.556303	1
0	0.158786	0.317946	0
0	0.038095	0.198538	0
0	0.038095	0.066080	0
0	0.038095	0.054139	0
0	0.038095	0.040894	0
0	0.767607	0.039700	1
1	2.217441	0.694935	2
2	2.002903	1.999666	2
